# Characterization of a Novel BmαTX47 Toxin Modulating Sodium Channels: The Crucial Role of Expression Vectors in Toxin Pharmacological Activity

**DOI:** 10.3390/toxins6030816

**Published:** 2014-02-26

**Authors:** Tian Li, Lingna Xu, Honglian Liu, Yawen He, Songping Liang, Wenxin Li, Yingliang Wu

**Affiliations:** 1 State Key Laboratory of Virology, College of Life Sciences, Wuhan University, Wuhan 430072, China; E-Mails: fiona2210@163.com (T.L.); xulingna58@126.com (L.X.); honglianliu2011@whu.edu.cn (H.L.); hesunrise@sohu.com (Y.H.); 2 Key Laboratory of Protein Chemistry and Developmental Biology of the Ministry of Education, College of Life Sciences, Hunan Normal University, Changsha 410081, Hunan, China; E-Mail: liangsp@hunnu.edu.cn

**Keywords:** *Buthus martensii* Karsch, BmαTX47, recombinant expression, sodium channels, pET-28a vector, pET-14b vector

## Abstract

Long-chain scorpion toxins with four disulfide bridges exhibit various pharmacological features towards the different voltage-gated sodium channel subtypes. However, the toxin production still remains a huge challenge. Here, we reported the effects of different expression vectors on the pharmacological properties of a novel toxin BmαTX47 from the scorpion *Buthus martensii* Karsch. The recombinant BmαTX47 was obtained using the expression vector pET-14b and pET-28a, respectively. Pharmacological experiments showed that the recombinant BmαTX47 was a new α-scorpion toxin which could inhibit the fast inactivation of rNa_v_1.2, mNa_v_1.4 and hNa_v_1.5 channels. Importantly, the different expression vectors were found to strongly affect BmαTX47 pharmacological activities while toxins were obtained by the same expression and purification procedures. When 10 µM recombinant BmαTX47 from the pET-28a vector was applied, the values of *I*_5ms_/*I*_peak_ for rNa_v_1.2, mNa_v_1.4 and hNa_v_1.5 channels were 44.12% ± 3.17%, 25.40% ± 4.89% and 65.34% ± 3.86%, respectively, which were better than those values of 11.33% ± 1.46%, 15.96% ± 1.87% and 5.24% ± 2.38% for rNa_v_1.2, mNa_v_1.4 and hNa_v_1.5 channels delayed by 10 µM recombinant BmαTX47 from the pET-14b vector. The dose-response experiments further indicated the EC_50_ values of recombinant BmαTX47 from the pET-28a vector were 7262.9 ± 755.9 nM for rNa_v_1.2 channel and 1005.8 ± 118.6 nM for hNav1.5 channel, respectively. Together, these findings highlighted the important role of expression vectors in scorpion toxin pharmacological properties, which would accelerate the understanding of the structure-function relationships of scorpion toxins and promote the potential application of toxins in the near future.

## 1. Introduction

Voltage-gated sodium channels (VGSCs) are essential for initiating and propagating action potentials in most electrically excitable cells of multicellular organisms [1]. VGSCs are transmembrane proteins consisting of a pore-forming α subunit and one or two auxiliary β subunits. So far, nine α-subunit isoforms (Na_v_1.1–Na_v_1.9) and five β subunit isoforms (β1B, β1–β4) have been identified in the vertebrates [1,2]. The α subunits are comprised of four homologous domains (DI-DIV), each containing six transmembrane helices (S1–S6) connected by extracellular and intracellular loops of variable sizes. So far, the conformational features of vertebrate VGSCs still remain unclear, and the structural complexity is often deciphered by the specific scorpion toxins and other animal toxins nowadays [3,4,5,6].

Scorpion toxins specific for the VGSCs are polypeptides containing 60–70 amino acids, stabilized by four disulfide bonds. Over the past years, these toxins have been proven invaluable to understand the complicated structural and functional features of the VGSCs [7]. Based on the different functions and mechanisms, the scorpion toxins specific for the VGSCs are mainly divided into two groups: α-toxins and β-toxins. The α-toxins delay the fast channel inactivation and have minor effects on the voltage-dependence of activation through binding to the sodium channel site 3 [8]. In contrast, the β-toxins hyperpolarize the activation curve and reduce the sodium peak current through binding to site 4 [7]. Although the α-toxins display the conserved tertiary structures, they show the remarkable diversity of sequence and function [8]. Thus, α-toxins are mainly divided into three subgroups: classical α-toxins predominantly acting on the mammalian VGSCs; insect α-toxins mainly acting on the insect VGSCs; and α-like toxins acting on both the mammalian and insect VGSCs. In order to investigate their pharmacological functions, α-toxins are mainly obtained nowadays by isolation from the scorpion venom and gene engineering. Due to the tiny amount of scorpion crude venom available, only several native α-scorpion toxins have been isolated, including LqhII, LqhIII and LqhαIT, *etc*. [9,10,11,12,13,14]. The alternative strategy is to obtain the recombinant toxins through gene engineering, such as LqhαIT, AaH2, Lqh II, BmKM1 and BmαTX14 [15,16,17,18,19,20]. Significantly different from the successful production of the recombinant short-chain scorpion toxins specific for the potassium channels [21,22,23], the α-scorpion toxins specific for the VGSCs are often difficult to produce due to some unknown factors. For example, the α-like toxin LqhIII can only be acquired in the form of the fusion peptide His-apamin-LqhIII rather than only LqhIII [24]. Nowadays, this complexity seriously hinders the development of structure-function relationships of both α-scorpion toxins and VGSCs.

Here, we reported a novel α-scorpion toxin BmαTX47 from the scorpion *Buthus martensii* Karsch, and investigated the effect of expression vector on toxin production and function. It was found that the recombinant BmαTX47 could be obtained using both expression vectors pET-14b and pET-28a with the same procedures, however, BmαTX47 from the different expression vectors showed the distinct activities on rNa_v_1.2, mNa_v_1.4 and hNa_v_1.5 channels. These findings highlighted the important role of expression vectors on scorpion toxin functions, which would improve the understanding of the structure-function relationships of scorpion toxins and accelerate toxin application as molecular tools and prospective drugs.

## 2. Results and Discussion

### 2.1. Cloning and Sequence Analysis of BmαTX47

**Figure 1:**
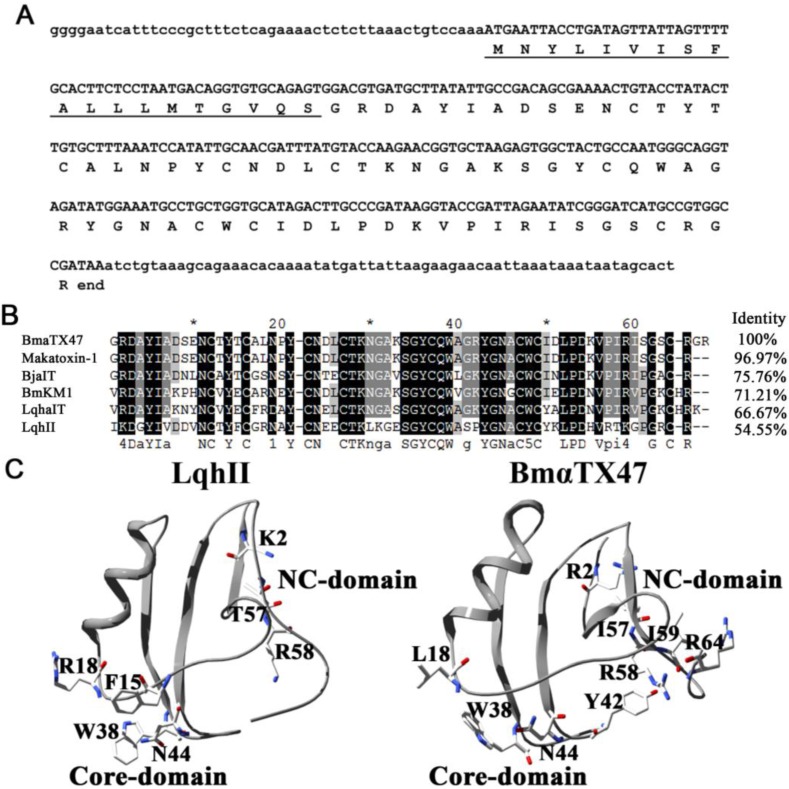
Sequence and structural analysis of the α-scorpion toxin BmαTX47. (**A**) The precursor nucleotide and amino acids sequences of BmαTX47. The signal peptide is underlined; (**B**) Amino acid sequence alignment of BmαTX47 with the known α-scorpion toxins including Makatoxin-1 (UniProtKB/Swiss-Prot: P56569.1), BmKM1 (UniProtKB/Swiss-Prot: P45697.2), BjαIT (UniProtKB/Swiss-Prot: Q56TT9.1), LqhII (UniProtKB/Swiss-Prot: P59355.1) and LqhαIT (UniProtKB/Swiss-Prot: P17728.2); (**C**) Structural modeling and analysis of BmαTX47. The experimental and putative functional residues in LqhII and BmαTX47 were labeled, respectively. The Lys2, Thr57 and Arg58 were functional residues composing the NC-domain, while the residues Phe15, Arg18, Trp38 and Asn44 constituted the Core-domain of LqhII. Accordingly, the residues Arg2, Ile57, Arg58, Ile59 and Arg64 were putative functional residues in NC-domain, while Leu18, Trp38, Tyr42 and Asn44 were possibly important residues in Core-domain of BmαTX47, respectively.

Through the random screening and bioinformatics analysis of the *B. martensii* venom gland cDNA library described previously [23,25,26,27], we found a new putative α-scorpion toxin BmαTX47 which might act on sodium channels. The precursor nucleotide sequence had 369 bp including a 5' untranslated region (UTR), ORF and 3' UTR of 48 bp, 258 bp and 63 bp, respectively. The ORF region encoded a polypeptide of 85 amino acid residues, containing a 19-residue signal peptide and a 66-residue mature peptide (Figure 1A).

Multiple sequence alignments showed that BmαTX47 shared high homology with other known α-scorpion toxins, including Makatoxin-1, BjαIT, BmKM1, LqhαIT, and LqhII (Figure 1B). Since there was no functional information of highly similar toxin Makatoxin-1 on the VGSC subtypes [28], it was essential to characterize the pharmacological function of BmαTX47 in this work. The structural modeling and analysis were performed using α-scorpion toxin BmKM1 (PDB code: 1ZYW) as the template. As shown in Figure 1C Bm αTX47 probably adopted the classical structure with an α-helix and three antiparallel β-sheets. Furthermore, the residues Arg2, Ile57, Arg58, Ile59, Arg64 and Arg66 were possible functional residues in the NC-domain, and residues Leu18, Trp38, Tyr42, Asn44 were possible functional residues in the Core-domain of the BmαTX47. Such distribution of potential functional residues resembled that of the classical toxin LqhII [15,16], which suggested that BmαTX47 was an α-scorpion toxin specific for the sodium channels.

### 2.2. Expression and Purification of BmαTX47 Using Expression Vector pET-14b

**Figure 2:**
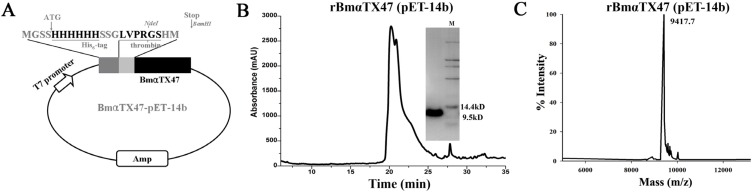
Expression, purification and identification of rBmαTX47 toxin with pET-14b vector. (**A**) Construction of expression vector BmαTX47-pET-14b. The nucleotide sequence encoding mature BmαTX47 peptide was inserted between NdeI and BamHI sites of the vector pET-14b downstream of the His_6_-tag and thrombin cleavage sequence. The full sequence of toxin rBmαTX47 was MGSSHHHHHHSSGLVPRGSHM-BmαTX47; (**B**) Purification of the refolded rBmαTX47 from pET-14b vector by RP-HPLC together with the tricine-SDS-PAGE analysis; (**C**) Mass spectrum of rBmαTX47 from the MALDI-TOF-MS. The calculated molecular weight was 9416.3 Da, and the measured value was 9417.7 Da.

During the production of the recombinant α-scorpion toxins, the expression vector pET-14b was ever used to express different toxins [15]. In this work, the expression vector pET-14b was first used to produce the recombinant BmαTX47. According to our previous method on the recombinant α-scorpion toxin BmαTX14 [20], BmαTX47 was inserted into pET-14b between NdeI and BmaHI sites, extending the N-terminus of BmαTX47 with a His_6_-tag and a thrombin cleavage site. The full sequence of the recombinant protein was MGSSHHHHHHSSGLVPRGSHM-GRDAYIADSENCTYTCALNPYCNDL CTKNGAKSGYCQWAGRYGNACWCIDLPDKVPIRISGSCRGR, with the first Met being excluded after translation (Figure 2A). The verified expression vector was subsequently transformed into *E. coli* Rosetta (DE3) for the toxin expression.

The recombinant BmαTX47 protein was expressed in the inclusion bodies, and was then denatured and refolded in 0.2 M ammonium acetate at 16 °C according to our previous procedure for the recombinant BmαTX14 toxin [20]. As shown in Figure 2B, the refolded toxin was separated by reverse-phase high performance liquid chromatography (RP-HPLC), and the peak at about 20 min corresponding to rBmαTX47 was manually collected and then analyzed by the sodium dodecyl sulfate polyacrylamide gel electrophoresis (SDS-PAGE) and MALDI-TOF-MS. It could be found that there was only one discernible band at about 9.5 kDa on the analytical gel (Figure 2B), and the determined molecular weight was 9417.7 Da, in accordance with the calculated molecular weight 9416.3 Da (Figure 2C. Subsequently, rBmαTX47 was quantiﬁed using the BCA Protein Assay kit (Thermo Fisher Scientiﬁc, Waltham, MA, USA) and stored at −80 °C after the freeze-drying. In previous cases of the recombinant α-scorpion toxins, the His_6_-tag was proven to have little effect on the toxin activities [15,20,24], so the tag was not removed in the fusion peptide His_6_-BmαTX47 in this work.

### 2.3. Pharmacological Characterization of rBmαTX47 from Expression Vector pET-14b

**Figure 3:**
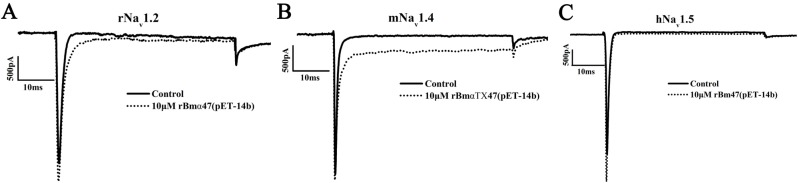
Pharmacological properties of rBmαTX47 on rNa_v_1.2, mNa_v_1.4 and hNa_v_1.5 channels. (**A**) rNa_v_1.2 current traces in the absence (control) or presence of 10 μM rBmαTX47 from the expression vector pET-14b; (**B**) mNa_v_1.4 current traces in the absence (control) or presence of 10 μM rBmαTX47 from the expression vector pET-14b; (**C**) hNa_v_1.5 current traces in the absence (control) or presence of 10 μM rBmαTX47 from the expression vector pET-14b.

The Na_v_1.2, Na_v_1.4 and Na_v_1.5 sodium channels are widely used to characterize the pharmacological profiles of α-scorpion toxins [9,10,15,17,29,30,31,32,33,34]. In this work, the pharmacological effects of rBmαTX47 from the expression vector pET-14b were also examined on the rNa_v_1.2, mNa_v_1.4 and hNa_v_1.5 channels expressed in HEK293 cells. As shown in Figure 3 and Table 1, the 10 µM rBmαTX47 could not efficiently inhibit the fast inactivation of all these channels and the *I*_5ms_/*I*_peak_ values were 11.33% ± 1.46% and 15.96% ± 1.87% for rNa_v_1.2 and mNa_v_1.4 channels, respectively. Meanwhile, the 10 μM rBmαTX47 was also found to have little effect on the currents of hNa_v_1.5 channel (Figure 3C and Table 1). These results indicated that the rBmαTX47 from the expression vector pET-14b was not a potent modulator for sodium channels examined above. However, whether toxin BmαTX47 was a potent modulator remained unclear.

In our previous work on the recombinant BmαTX14, the different pharmacological activities by different expression vectors were observed. The recombinant BmαTX14 expressed in *Pichia pastoris* could block sodium channel currents without affecting the gating kinetics in mouse trigeminal root ganglion neurons [35]. However, the recombinant BmαTX14 produced by the expression vector pET-28a could inhibit the fast inactivation of mNa_v_1.4 sodium channels with the EC_50_ of 82.3 ± 15.7 Nm [20]. Importantly, both pET-14b and pET-28a vectors are sometimes used for the expression of different α-scorpion toxins [15,20,24]. These developments suggested the potential effects of the expression vectors and production strategies on toxin activities, and prompted us to further investigate the pharmacological properties of rBmαTX47 using the expression vector pET-28a.

**Table 1:** Effects of rBmαTX47 on rNa_v_1.2, mNa_v_1.4 and hNa_v_1.5 channels. Each value represents mean ± SE (*n* ≥ 3).

Toxins (10 μM)	*I*_5ms_/*I*_peak_ on rNa_v_1.2 (%)	*I*_5ms_/*I*_peak_ on mNa_v_1.4 (%)	*I*_5ms_/*I*_peak_ on hNa_v_1.5 (%)
rBmαTX47 (pET-14b)	11.33 ± 1.46	15.96 ± 1.87	5.04 ± 2.38
rBmαTX47 (pET-28a)	44.12 ± 3.17	25.40 ± 4.89	65.34 ± 3.86

### 2.4. Preparation and Pharmacological Features of rBmαTX47 Using Expression Vector pET-28a

**Figure 4:**
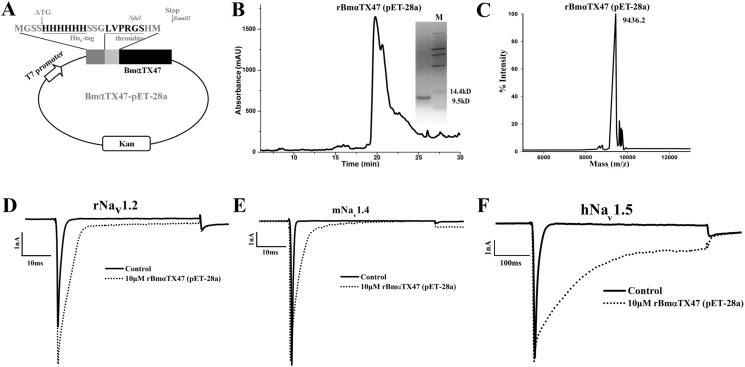
Preparation and pharmacological features of rBmαTX47 from pET-28a vector. (**A**) Construction of expression vector BmαTX47-pET-28a. The nucleotide sequence encoding mature BmαTX47 peptide was inserted between NdeI and BamHI sites of the vector pET-28a downstream of the His_6_-tag and thrombin cleavage sequence. The full sequence of toxin rBmαTX47 was MGSSHHHHHHSSGLVPRGSHM-BmαTX47; (**B**) Purification of rBmαTX47 from pET-28a vector by RP-HPLC together with the tricine-SDS-PAGE analysis; (**C**) Mass spectrum of rBmαTX47 from the MALDI-TOF-MS. The calculated molecular weight was 9416.3 Da, and the measured value was 9436.2 Da; (**D**) rNa_v_1.2 current traces in the absence (control) or presence of 10 μM rBmαTX47 produced from the expression vector pET-28a; (**E**) mNa_v_1.4 current traces in the absence (control) or presence of 10 μM rBmαTX47 produced from the expression vector pET-28a; (**F**) hNa_v_1.5 current traces in the absence (control) or presence of 10 μM rBmαTX47 produced from the expression vector pET-28a.

According to the toxin production procedure above, rBmαTX47 was also successfully obtained using the expression vector pET-28a, with the identical sequence to the toxin from the expression pET-14b (Figure 4AB, the peak at 20 min corresponding to the refolded rBmαTX47 was collected manually during the RP-HPLC separation and then analyzed by the SDS-PAGE, which showed only one discernible band at about 9.5 kDa (Figure 4B). The MALDI-TOF-MS showed the molecular weight of 9436.2 Da, which was a little different from the calculated 9416.3 Da, probably due to some post-translational processing (Figure 4C). Different from the activity of rBmαTX47 from the expression vector pET-14b, rBmαTX47 from the expression vector pET-28a was more potent for rNa_v_1.2, mNa_v_1.4 and hNa_v_1.5 channels. When 10 µM rBmαTX47 from pET-28a was applied, the fast inactivation of sodium channel currents was inhibited, with the *I*_5ms_/*I*_peak_ values of 44.12% ± 3.17%, 25.40% ± 4.89% and 65.34% ± 3.86% for rNa_v_1.2, mNa_v_1.4 and hNa_v_1.5 channels, respectively (Figure 4D–F, Table 1). These data showed that the expression vector pET-28a could improve the activity of the scorpion toxin rBmαTX47.

### 2.5. The Pivotal Role of Expression Vectors in Scorpion Toxin Pharmacological Properties

**Figure 5:**
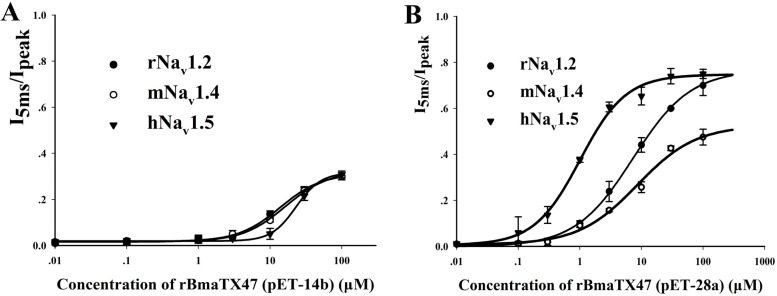
Pharmacological properties of rBmαTX47 from the different expression vectors on rNa_v_1.2, mNa_v_1.4 and hNa_v_1.5 channels. (**A**) Dose-response curves of rBmαTX47 (pET-14b) on rNa_v_1.2, mNa_v_1.4 and hNa_v_1.5 channels; (**B**) Dose-response curves of rBmαTX47 (pET-28a) on rNa_v_1.2, mNa_v_1.4 and hNa_v_1.5 channels. Data represent the mean ± SE of at least three independent experiments.

In order to further investigate the effect of the expression vector on the pharmacological activities of rBmαTX47 toxin, the dose-response of rBmαTX47 was conducted thereafter. For rBmαTX47 toxin from the expression vector pET-14b, 100 μM toxins displayed the little selectivity and weak potency among rNa_v_1.2, mNa_v_1.4 and hNa_v_1.5 channels, with the *I*_5ms_/*I*_peak_ values of about 30% for all these channels (Figure 5A). However, the 100 μM rBmαTX47 toxin from the expression vector pET-28a could more significantly inhibit the fast inactivation of rNa_v_1.2 with *I*_5ms_/*I*_peak_ value of about 70%. The corresponding EC_50_ value was 7262.9 ± 755.9 nM through fitting with the Hill equation (Figure 5B). As for the mNa_v_1.4 channel, expression vector pET-28a also improved the activity of rBmαTX47, and *I*_5ms_/*I*_peak_ value raised to about 50% for mNa_v_1.4 channel in the presence of 100 μM rBmαTX47 (Figure 5B). Besides, the 100 μM rBmαTX47 from the expression vector pET-28a could efficiently delay the fast inactivation of hNa_v_1.5 with the *I*_5ms_/*I*_peak_ value of about 75%, and the corresponding EC_50_ value was 1005.8 ± 118.6 nM. Importantly, it could be noted that rBmαTX47 showed preference for rNa_v_1.2 and hNa_v_1.5 channels to mNa_v_1.4 channel, which usually happened for the classical α-toxins predominantly acting on the mammalian VGSCs [9,10,15,16,29,30,31,34,36]. Overall, the different activities and selectivities of rBmαTX47 from the expression vectors pET-14b and pET-28a indicated the critical role of the expression vectors in the scorpion toxin pharmacological properties. In addition, the pharmacological activities of rBmαTX47 further supported the function of the nearly identical Makatoxin-1 (Figure 1B), which exhibited nitrergic action in rat anococcygeus muscle expressing Na_v_1.4 and Na_v_1.5 channels [28,37].

So far, the different expression vectors were used to produce α-scorpion toxins, and their effects on the toxin pharmacology were not systematically investigated. Although the pET-14b and pET-28a vectors were often used for α-scorpion toxins’ expression, the unknown factor affecting toxin expression was also observed. For example, the pET-14b was used to express the classical α-scorpion toxin LqhII and its mutants [15,16,32]. However, the α-like toxin LqhIII was difficult to obtain by the pET-14b vector until extended by an 18-resiude apamin peptide as a linker [24]. Such phenomenon was also confirmed by our group (unpublished data). The vector pET-28a was employed for the production of the α-scorpion toxins BmαTX14 [20], BmKIT3 [38], MeuNaTxα-12 and MeuNaTxα-13 [39]. Besides the expression vector pET-14b and pET-28a, other vectors were also used to express α-scorpion toxins, such as the pET-11cK vector for expressing LqhαIT [13,32,40,41] and LqhIT2 [42], and the pVT 102 U/α vector for BmKM1 and its mutants [18,19,43]. Significantly different from the successful expression of many scorpion toxins (with 30~40 residues) specific for the potassium channels by the pGEX-6p-1 vector [21,22,23], these various vectors suggested the huge difficulty in expressing α-scorpion toxins due to some unknown factors. Therefore, the greater effort is critical for expressing the same α-scorpion toxins by different expression vectors in the near future. These efforts would undoubtedly accelerate toxin research and potential application as the molecular tools and prospective drugs.

## 3. Experimental Section

### 3.1. cDNA Library Construction and Screening

The venom gland cDNA library of the scorpion *B. martensii* was constructed as described previously [25,44,45]. Random colonies were selected for sequencing using the ABI 3730 automated sequencer. Open reading frames (ORFs) of the sequences were characterized using the ORF Finder (NCBI, Bethesda, MD, USA, http://www.ncbi.nlm.nih.gov/projects/gorf/). Signal peptides were removed using the SignalP 4.0 Server (Center for Biological Sequence Analysis, Lyngby, Denmark, http://www.cbs.dtu.dk/services/SignalP/). All sequence alignments were performed using GeneDoc software (National Resource for Biomedical Supercomputing, Pittsburgh, PA, USA) followed by manual adjustment. Sequences of α-scorpion toxins were obtained by searching our cDNA libraries and the GenBank National Center for Biotechnology Information database (NCBI, Bethesda, MD, USA) using the Basic Local Alignment Search Tool algorithm.

### 3.2. Materials

Expression plasmids containing the cDNA encoding the rat brain sodium channel α-subunit rSCN2A (pNaG_2_), the mouse skeletal muscle sodium channel α-subunit mSCN4A (pcDNA3.0-mH_2_) and the human heart sodium channel α-subnit hSCN5A (pCDNA3.1(+)-hH_1_)were kindly provided by Dr. Alan L. Goldin (University of California, Irvine, CA, USA), Dr. Thomas Zimmer (Friedrich Schiller University of Jena, Jena, Germany) and Dr. Songping Liang (Hunan Normal University, Hunan, China), respectively.

### 3.3. Construction of Expression Vectors

We used the cDNA sequence of BmαTX47 from the scorpion *B. martensii* venom glands cDNA library as the template for polymerase chain reaction (PCR). The PCR product was digested with NdeI and BamHI and inserted into the expression vector pET-14b and pET-28a, respectively. After verification through DNA sequencing, the plasmids BmαTX47 from both vectors were transformed into *Escherichia coli* Rosetta (DE3) cells for expression.

### 3.4. Expression and Purification of BmαTX47

To obtain recombinant BmαTX47, cells with the expression plasmids BmαTX47 (pET-14b) and BmαTX47 (pET-28a) were incubated and induced with 0.5 mM IPTG in LB medium as previously described [20]. Recombinant BmαTX47 protein was expressed in inclusion bodies and then denatured and refolded in 100-fold volume of 0.2 M ammonium acetate at 16 °C. The refolded protein was ultrafiltered and puriﬁed by HPLC on a C18 column (10.0 mm × 250 mm, 5 μm; Elite-HPLC, Dalian, China). The fraction containing recombinant BmαTX47 was eluted 20–21 min after injection and was further analyzed by matrix-assisted laser desorption ionization time-of-ﬂight mass spectrometry (MALDI- TOF-MS; Voyager-DESTR, Applied Biosystems, Foster City, CA, USA).

### 3.5. Electrophysiology

HEK293 cells were maintained in Dulbecco’s modiﬁed Eagle’s medium (Gibco, Grand Island, NY, USA), supplemented with 10% heat-inactivated fetal calf serum, 100 U/mL ampicillin, and 100 μg/mL streptomycin, in a 5% CO_2_ incubator at 37 °C. They were transiently transfected with a 1:2 ratio of the sodium channel expression plasmids and a vector encoding enhanced green ﬂuorescent protein (EGFP) using the TurboFect *in vitro* Transfection Reagent (Thermo Scientific, Pittsburgh, PA. USA). Sodium channel currents were recorded 2 days afterwards in the EGFP-positive cells.

Whole-cell patch-clamp experiments were performed at 25 °C. Data were recorded with an EPC-10 patch-clamp ampliﬁer (HEKA Elektronik, Lambrecht, Germany) interfaced to a computer running acquisition and analysis software Pulse (HEKA Elektronik, Lambrecht, Germany). The patch pipettes contained 35 mM NaCl, 105 mM CsF, 10 mM EGTA, and 10 mM HEPES (pH adjusted to 7.4 with CsOH). The bath solution contained 150 mM NaCl, 2 mM KCl, 1.5 mM CaCl_2_, 1 mM MgCl_2_, 2 mM Na_2_ATP, and 10 mM HEPES (pH adjusted to 7.4 with NaOH). Peptides were dissolved in the bath solution containing 1% bovine serum albumin and then applied at above-mentioned concentrations.

To measure toxin-induced inhibition of fast inactivation, currents were elicited by depolarizing to 0 mV from the holding potential of −100 mV. Both the peak currents and the mean currents between 4.5 and 5 ms after depolarization were recorded in the absence and the presence of BmαTX47 peptides. The ratio *I*_5ms_/*I*_peak_ indicates the proportion of active sodium channels whose necessary conformational changes for fast inactivation were impaired by the toxin. The concentration dependence of this toxin-induced inhibition was calculated by plotting *I*_5ms_/*I*_peak_ as a function of toxin concentrations and ﬁtted with the Hill equation as follows:

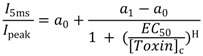
(1)


Here, H is the Hill coefficient; *a*_1_ − *a*_0_ represents the recorded maximal effect of toxin-induced inhibition at saturating concentrations; *a*_0_ is the measured value before applying the toxin; [*Toxin*]_c_ is the toxin concentration; and *EC*_50_ represents the toxin concentration at the half-maximal effect. Data analysis was performed using SigmaPlot. All data are shown as arithmetic mean ± SE.

## 4. Conclusions

In this work, we cloned and expressed a novel α-scorpion toxin rBmαTX47 from the scorpion *Buthus martensii* Karsch. The two expression vectors pET-14b and pET-28a were found to remarkably affect the toxin pharmacological activities. The toxin rBmαTX47 from the vector pET-14b slightly inhibited the fast inactivation of rNa_v_1.2, mNa_v_1.4 and hNa_v_1.5 channels; however, rBmαTX47 from the pET-28a vector significantly inhibited the fast inactivation of these three sodium channels. For rNa_v_1.2 and hNav1.5 channels, the *EC*_50_ values of rBmαTX47 from the pET-28a vector were 7262.9 ± 755.9 nM and 1005.8 ± 118.6 nM, respectively, which were more potent than those of toxin from the pET-14b. Besides the different activities, the selectivity of rBmαTX47 was also influenced by the expression vectors. The toxin rBmαTX47 from pET-28a vector showed preference for rNa_v_1.2 and hNa_v_1.5 channels to mNav1.4 channel, while the toxin from pET-14b vector showed little selectivity among the three sodium channel isoforms. These findings not only highlighted the important role of expression vectors on scorpion toxin function, but would also accelerate studies on gene engineering and future applications of scorpion toxins acting on sodium channels.
